# The Use of Flowable Decellularized Human Placental Connective Tissue Matrix in Alveolar Ridge Preservation: A Split-Mouth Pilot Study

**DOI:** 10.3390/dj13110545

**Published:** 2025-11-20

**Authors:** Bachar Husseini, Ronald Younes, Nabil Ghosn, Robert Miller, Georges Khoury, Robert Hariri, Michel Dard

**Affiliations:** 1 Private Practice, Beirut 17-5208, Lebanon; 2 Department of Oral Surgery, Saint Joseph University of Beirut, Beirut 17-5208, Lebanon; 3 Department of Digital Dentistry, Saint Joseph University of Beirut, Beirut 17-5208, Lebanon; 4 Miller and Korn Periodontics and Implant Solutions, Plantation, FL 33324, USA; 5 Department of Advanced Surgical Implantology, Service of Odontology, U.F.R. of Odontology, Rothschild Hospital, AP-HP, University Denis Diderot, 75010 Paris, France; 6 Research & Development, Degenerative Diseases, Celularity Inc., 170 Park Ave., Florham Park, NJ 07932, USA; 7 Section of Oral, Diagnostic and Rehabilitation Sciences, College of Dental Medicine, Columbia University, New York, NY 10032, USA

**Keywords:** alveolar ridge augmentation, bone regeneration, cone-beam computed tomography, extracellular matrix, tooth extraction, wound healing

## Abstract

**Background/Objectives:** Tooth extraction is known to cause both bone loss and soft tissue collapse, changes that can complicate implant placement. While alveolar ridge preservation techniques have been proposed to limit these alterations, they often fail to maintain both hard and soft tissue dimensions at the same time. Placental-derived extracellular matrices offer a biologically active adjuvant, providing structural proteins that may support healing. The purpose of this study was to assess whether a flowable decellularized Human Placental Connective Tissue Matrix (HPCTM), combined with an allogeneic bone substitute, could improve ridge preservation by addressing changes in soft tissue as the primary outcome and underlying bone volume as the secondary outcome. **Methods:** In a split-mouth, randomized pilot trial, hopeless teeth in opposite quadrants were atraumatically extracted. Test sockets were grafted with allograft mixed with HPCTM, while control sockets received allograft alone. Healing was followed clinically and digitally using intra-oral scans; standardized photographs at 10, 21, and 30 days post-operatively; and cone-beam computed tomography at 4 months post-operatively. **Results:** Ten patients completed the study (10 test sites and 10 control sites). Sites treated with HPCTM showed faster and more stable healing. Gingival shrinkage was consistently reduced at test sites, with Hodges–Lehmann median differences of 0.50 mm at Day 10 (95% CI: 0.29–0.62; *p* = 0.0039), 0.54 mm at Day 21 (95% CI: 0.42–0.65; *p* = 0.002), and 0.54 mm at Day 30 (95% CI: 0.39–0.68; *p* = 0.002). Radiographically, test sites lost significantly less bone volume (28.24 ± 2.43%) compared with controls (38.85 ± 1.29%; *p* = 0.019). **Conclusions:** Within the limits of this study, HPCTM appears to support better preservation of both gingival architecture and alveolar bone after extraction.

## 1. Introduction

Following tooth extraction, the alveolar bone is subject to physiological resorption that could take up to 50% of its volume in the first year post-extraction [1]. This dimensional collapse can compromise prosthetically driven implant placement [2]. Many surgical procedures have been proposed to reduce this physiological occurrence, such as socket shield therapy [3], immediate implant placement via the dual zone concept, and diverse alveolar ridge preservation techniques [4].

Alveolar ridge preservation (ARP) is a surgical approach that could be applied when immediate implant therapy cannot be performed; it consists of filling the socket with a bone substitute, whether it is allogenic, xenogeneic, or alloplastic, and sealing it with a barrier to assure undisturbed healing and reduce bacterial and epithelial colonization of the graft [5].

Currently, the literature reports on numerous methods of socket closure by the use of a collagen membrane, free gingival graft, Polytetrafluoroethylene membrane, or soft tissue substitute like collagen matrices [6]. However, the resorption of collagen-derived materials is considered to be uncontrollable and can leave the graft material exposed in the early phases of healing [7]. Additionally, the amount of keratinized tissue loss remains a serious threat to the future implant site [8]. In fact, only a few articles have investigated soft tissue shrinkage following alveolar ridge preservation [9,10,11]. It seems that no specific socket sealing technique can yield satisfying results to prevent the observed collapse. Concerning hard tissue resorption, it has been proven that despite socket grafting, bone resorption will take place. This can jeopardize implant placement in delicate cases such as the anterior sector.

Due to the aforementioned drawbacks, clinicians and researchers have started to explore more biological ways to enhance the healing process and reduce soft tissue shrinkage and hard tissue resorption together while simplifying the surgical procedure. Recently, the American Academy of Periodontology (AAP) published a paper concerning the application of biologic adjuvants in oral surgery. According to this AAP best-evidence paper, these adjuvants can accelerate the healing process by attracting essential growth factor synthesis through chemotaxis [12]. Due to their abundance in extracellular matrix components such as collagen, elastin, and proteoglycans, human placental derivatives are considered one of these biological adjuvants that could be used in oral surgeries [13,14,15]. Each part of the placenta yields a specific composition that differs in terms of function, usability, and preservation [16,17]. Among the placental components rises the chorionic plate, which is composed of a thick layer of connective tissue [16,17]. Biologically, it is composed of elastin and collagen types I and III in addition to proteoglycans such as laminin and fibronectin [17]. In order to extract the usable chorion, donated human placentas obtained from normal, healthy, and full-term pregnancies are subjected to a series of controlled scraping and washing to remove viable cells from the substance of the tissue [17]. Once washed, the chorionic plate is homogenized into a paste and lyophilized; then, it is ground into connective tissue matrix powder and filled into vials or syringes. The finished product is an allogeneic particulate HPCTM devoid of cells, hormones, growth factors, and cytokines [17]. Decellularization removes cells and most soluble mediators to improve biocompatibility; however, it does not eliminate the extracellular matrix. The preserved matrix retains collagen- and fibronectin-based integrin-binding sites, laminins, and glycosaminoglycans that provide structural and biochemical cues. In extraction sockets, such scaffolds can support clot stability, guide cell migration, and modulate the early inflammatory response, processes that together may lessen soft tissue collapse and ridge contraction.

On this basis, adjunctive use of a flowable placenta-derived decellularized connective tissue matrix (HPCTM) with allograft may provide a provisional matrix to support healing even in the absence of cells, hormones, or added growth factors [17].

To the best of the authors’ knowledge, no prior report has evaluated a flowable HPCTM in alveolar ridge preservation or its effect on post-extraction tissue collapse. This randomized split-mouth pilot study compared allograft plus flowable HPCTM with allograft alone, assessing linear soft tissue change at Days 10, 21, and 30 (primary outcome) and cone-beam computed tomography (CBCT)-measured volumetric bone resorption at 4 months (secondary outcome).

## 2. Materials and Methods

### 2.1. Study Design, Randomization, and Ethics

This study was a split-mouth, prospective, randomized pilot study. The study protocol was approved by the university institutional review board (USJ-2024-175) on 9 July 2024 and registered at the Clincaltrials.gov (https://www.equator-network.org/reporting-guidelines/consort-2010-statement-extension-to-randomised-pilot-and-feasibility-trials/) accessed on 23 August 2024 using the following number: NCT06915675. The study followed the CONSORT extension guidelines for pilot and feasibility studies.

Patient enrolment started in August 2024 and ended in January 2025. Enrolled patients were informed about the product and the surgical intervention in detail and signed an informed consent stating their right to quit the study at any time point in respect of the Declaration of Helsinki on medical research and allowing the authors to use the recorded data in scientific research.

Surgical sites were allocated to test or control groups based on a coin toss model; neither the surgeon nor the results investigator could be blinded due to the nature of the study.

The inclusion and exclusion criteria are presented in Table 1.

### 2.2. Surgical Intervention

**Figure 1:**
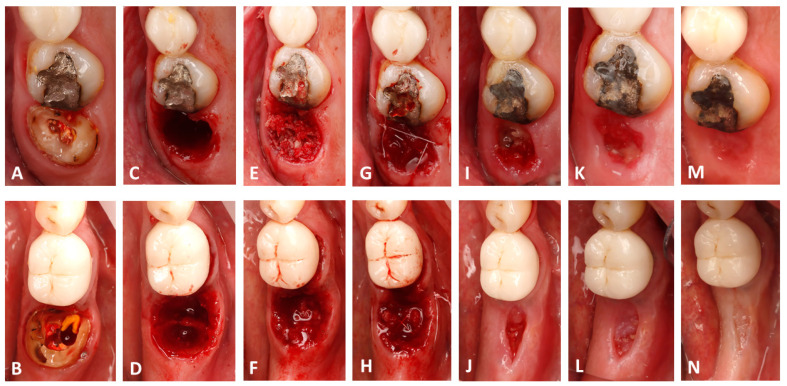
Clinical photographs showing the surgical workflow and the follow-ups; note that the upper row presents the control site while the lower row presents the test site. (**A**,**B**) The pre-operative state. (**C**,**D**) Socket status after extraction. (**E**,**F**) Grafting the socket; note the stable hemostatic film on the first molar. (**G**,**H**) Tension-free sutures. (**I**,**J**) The surgical site at 10 days. (**K**,**L**) The surgical site at 21 days. (**M**,**N**) The surgical sites at 30 days post-op.

**Figure 2:**
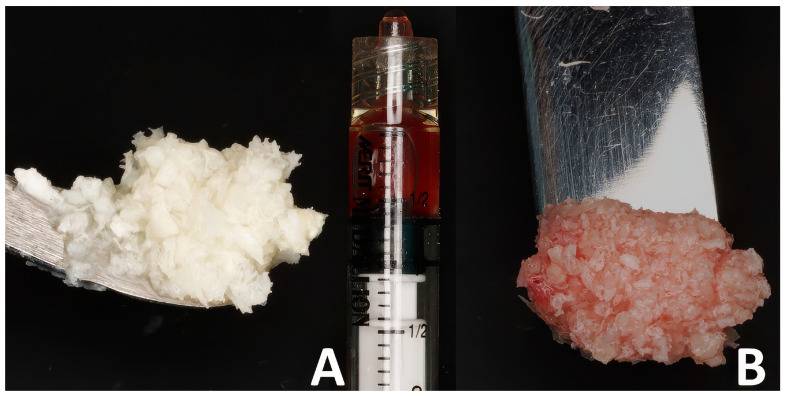
The bone putty preparation steps: (**A**) allogeneic bone chips and the prepared serum; (**B**) the moldable putty.

All surgeries were performed by a single operator (B.H.). Following 4%Articaine-based local anesthesia administration (Septanest, Septodont, Île-de-France, France) and crown removal if one was present (Figure 1A,B), a minimally invasive cut using fissure carbide burrs (FG701, Neoburr, Republic of Korea) was performed to separate the mesial and distal roots. Subsequently the roots were luxated and extracted using elevators (EL3S, Hu-Friedy, Chicago, IL, USA) (Figure 1B,C). The alveolar sockets were debrided using Lucas curettes (Hu-Friedy, Chicago, IL, USA). Since avoiding blood inundation of the bone chips was not possible and to avoid any methodology bias, allografts were mixed with blood collected from the extraction socket after debridement at both sites. The test sites received 0.5 cc of decellularized HPCTM (Orafyl, Biocellgraft, NewYork, NY, USA), regulated by the US Food and Drug Administration (FDA) under Section 361 of the Public Health Service (HCT/P, 21 CFR, Part 1271.10(a)), mixed with 0.5 cc of the patient’s blood; the mixing procedure involved the use of a double-syringe model with a mixing time of 2 min. A volume of 1 cc of allograft particles (Maxgraft Cortico cancellous granules, Botiss, Germany) was mixed with the prepared serum (Figure 2A) continuously till a moldable putty was formed (Figure 2B). Subsequently, the bone putty was inserted into the socket and compacted softly till the radiographic coronal limit of the socket was reached (Figure 1F). Due to the compaction process, the excess blood mixed with the gel filled the gingival portion of the socket, resulting in a thick gelatinous seal of the socket (Figure 1F). X-shaped 5/0 Polytetrafluoroethylene sutures (Biotex, Purgo biologics, Republic of Korea) were placed on top of the socket to secure the grafted particles from spilling; care was taken to not displace the gingival margins of the socket (Figure 1H). The control socket received the same therapeutic treatment but with blood solely mixed into the allografts (Figure 1E) and a 2.5 × 7.5 cm collagen fleece tailored to the socket orifice perimeter (Collatape, Zimvie, Palm Beach Gardens, FL, USA) sutured on top to prevent the bone particles from being displaced (Figure 1G).

As a post-operative recommendation, the patients were advised to abstain from any gargling or spitting for 24 h. The patients were given antibiotics for 7 days at 2 g/day orally (Amoxicillin, Sandoz, Switzerland), the non-steroidal anti-inflammatory drug ibuprofen 400 mg (Abbott Laboratories, Chicago, IL, USA) three times daily for 3 days, and a 0,12% chlorhexidine mouthwash (Paroex, Sunstar, Switzerland) three times daily for 2 weeks.

**Figure 3:**
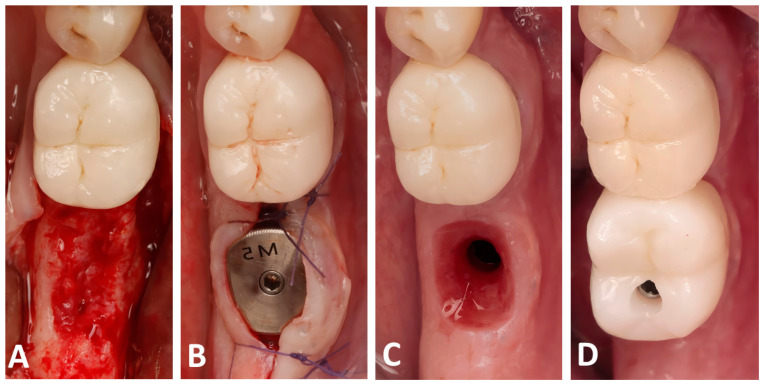
Clinical photographs showing the implant placement and the prosthetic placement: (**A**) the alveolar bone following full-thickness flap elevation; (**B**) implant placement with an anatomical healing abutment; (**C**) the created emergence profile; (**D**) screw-retained zirconia crown delivery.

At 4 months post-op, a CBCT scan was performed to assess the bone dimensions, and patients were recalled for implant placement. A full-thickness flap was raised (Figure 3A) and implants (Blue diamond, Mega’gen, Republic of Korea) were placed in a prosthetically driven fashion, making sure that the fixture was surrounded by at least 2 mm of bone buccally and lingually. Anatomical healing caps (Mega’gen, Republic of Korea) were used in order to shape an optimal prosthetic emergence profile (Figure 3B). Finally, two months later, a digital impression of the placed implant was performed using an intra-oral scan (R2I3, Mega’gen, Republic of Korea), and screw-retained zirconia crowns were delivered for each patient (Figure 3C,D).

### 2.3. Data Collection

The data collection process was divided into two phases: pre-ARP and post-ARP. Pre-operatively, each patient underwent CBCT and intra-oral scanning; all data were collected and stored separately for each patient in secure hard drives. Post-operatively, the patients returned at 10, 21, and 30 days for clinical review, intra-oral scans, and standardized clinical photographs taken using a digital single-lens reflex camera (Canon 90D, 100 mm macro lens, Tokyo, Japan) equipped with a light diffuser (Flash-Kap, Boston, MA, USA). Photographic acquisition was standardized (tripod stabilization, fixed exposure and focal length, and constant camera-to-subject distance and angulation, in addition to uniform lighting) to enable repeatable, unbiased assessments across visits. At 4 months post-op, patients were recalled for follow-up CBCT to plan implant placement.

### 2.4. Studied Parameters and Null Hypothesis Identification

The studied parameters were as follows:

1. Linear soft tissue shrinkage expressed in millimeters;
2. The volumetric bone resorption rate expressed as a percentage;
3. Clinical observation and interpretation of wound healing in the test and control groups.

In this randomized split-mouth pilot study, the null hypothesis stated that no within-subject difference would exist between the test intervention (allograft + flowable HPCTM) and the control (allograft alone) for the pre-specified endpoints: linear soft tissue change at Days 10, 21, and 30 (primary outcome) and CBCT-measured volumetric bone resorption at 4 months (secondary outcome).

### 2.5. Soft Tissue Healing Measurements

To achieve accurate measurements, several steps were followed. Firstly, all intra-oral scans were exported as a stereolithography file (STL). The initial intra-oral scans were imported into imaging software and aligned to the CBCT scan using multiple stable landmarks; this scan was set as a reference. Then the scans were exported to design software (Medit Design, Medit, Seoul, Republic of Korea) where the teeth in question were extracted virtually and the sockets filled to the top of the free gingiva. The follow-up intra-oral scans were imported into the same software and super-imposed using a semi-automatic option where up to 5 points were used as a fixed reference. The region of interest was delimited using the trimming option as follows:

- Mesio-distally: A distance of 1.5 mm from the relevant papilla peak;
- Apically: A bucco-lingual/palatal line tangent to the visible muco-gingival line and parallel to the occlusal surface.

**Figure 4:**
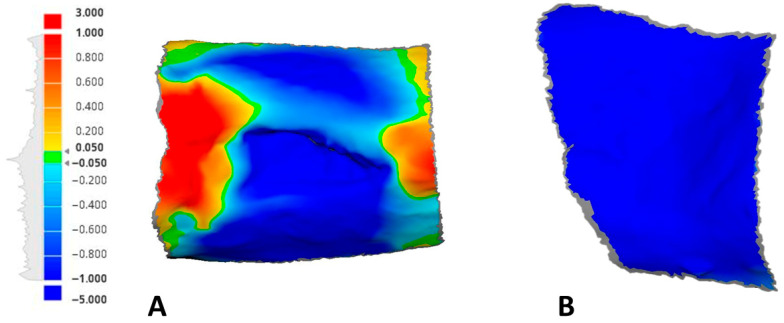
An example of the generated color maps with a unified scale measurement tool showing the soft tissue fluctuations at 21 days compared to the baseline: (**A**) test site; (**B**) control site.

Using the deviation analysis option of the software, a color map quantifying any positive or negative deviations between the different time point scans in comparison to the baseline was generated (Figure 4).

### 2.6. Bone Resorption Measurements

The hard tissue resorption measurement technique in a previous article published by the same authors was adopted [18]. Semi-automatic segmentation software (ITK-Snap 4.0, U.S. National Institute of Biomedical Imaging and BioEngineering, Bethesda, MD, USA) was used to load and segment the pre and post CBCT scans. First, both CBCTs were oriented according to the Frankfort plane, then aligned using multiple fixed landmarks in different orientations, ensuring a perfect match and easy reproducibility.

A standard region of interest was applied for all the measured sites as follows:

- Mesio-distal limit: A distance of 1.5 mm away from the mesial and distal surfaces of adjacent teeth.
- Bucco-lingual limit: Limited to the observed bone dimensions.
- Apico-coronal limit: Coronally, the most detectable bone peak on the mesial and distal sides; apically, 1.5 mm below the root tip.

**Figure 5:**
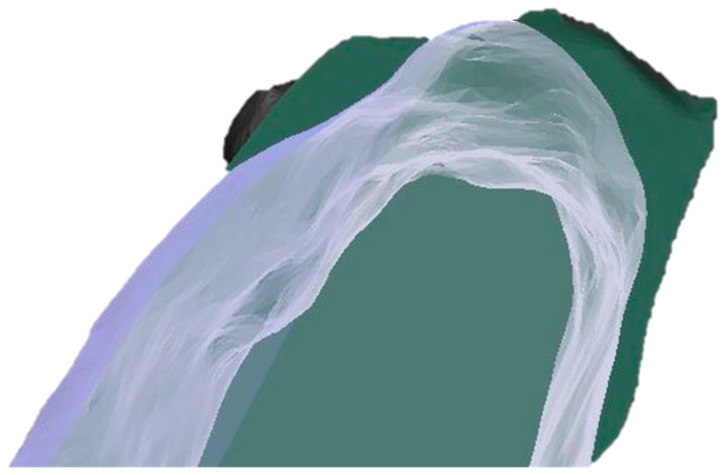
A visual example of the generated CBCT superimposition showing a comparison of the baseline and 4 months post alveolar ridge preservation at the test site (dark green, initial state; transparent white, 4 months post-op).

Using a pixel-based scale, a specific threshold value was used to select and separate the desired structure from its surroundings on both the pre- and post-surgery CBCT. Following the automatic segmentations, a manual inspection was applied to eliminate any undesired selections, and the volume of each created shape was measured using the software’s built-in tools (Figure 5).

The resorption rate was calculated using the following mathematical equation:

[(VPre-op − Vpost-op)/VPre-op] × 100.

### 2.7. Statistical Analysis

Given the split-mouth design and the limited sample size (n = 10 pairs), normality of the paired differences could not be reliably assumed. Comparisons of soft tissue shrinkage (mm) at Days 10, 21, and 30 between test and control sites were therefore conducted using the Wilcoxon signed-rank test. The results are presented as median paired differences with corresponding Hodges–Lehmann (HL) 95% confidence intervals, and *p*-values were adjusted across the three time points using the Holm–Bonferroni method. Effect sizes were expressed as the rank-biserial correlation. As a sensitivity analysis, paired permutation tests and paired *t*-tests were also performed; all approaches yielded a consistent direction and magnitude of effects. Volumetric bone resorption (%) was analyzed using the Wilcoxon signed-rank test. Statistical significance was set at α = 0.05 (two-sided). All analyses were conducted with IBM SPSS Statistics v25.

## 3. Results

**Figure 6:**
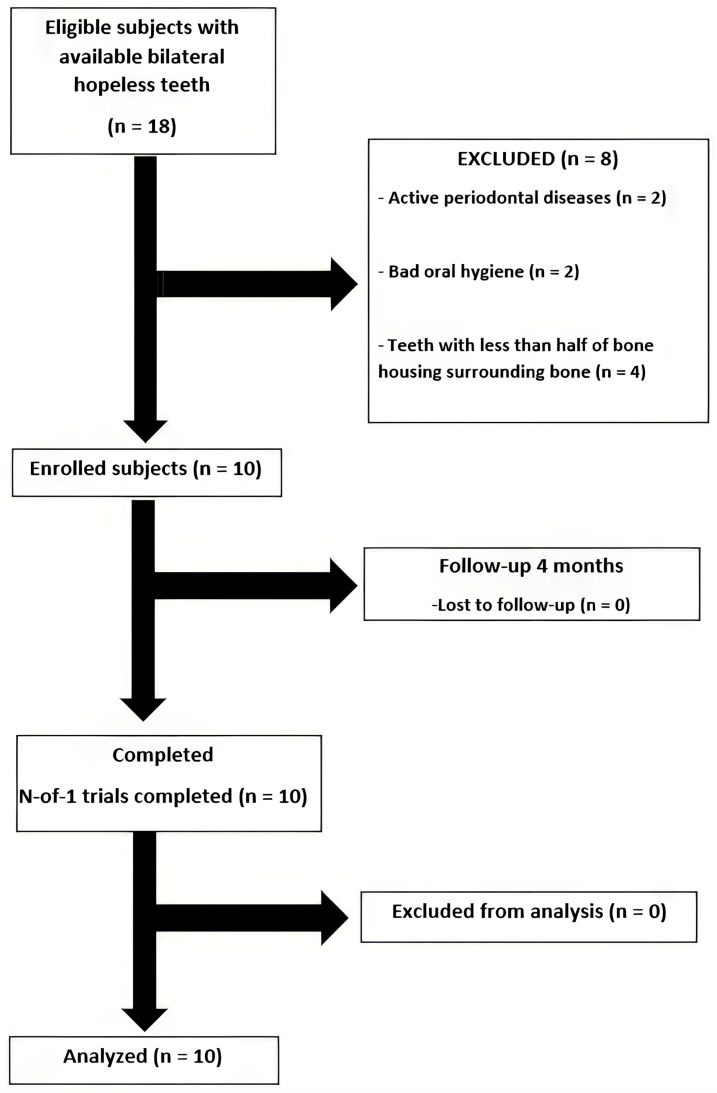
A flow chart of the studied population.

Eighteen patients were willing to participate in this study; ten patients passed the inclusion criteria screening and were enrolled into this study (Figure 6). The demographic data are shown in Table 2. All patients completed follow-ups without adverse events. Healing was uneventful in both groups, and no post-operative complications were observed.

### 3.1. Soft Tissue Shrinkage

**Figure 7:**
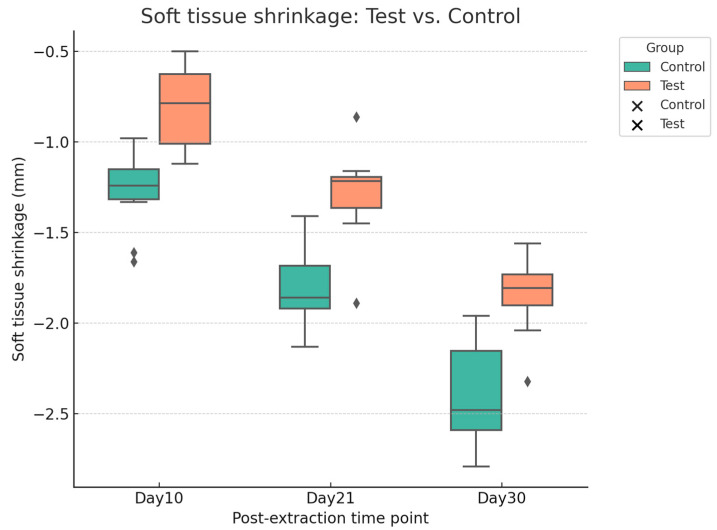
Boxplot with scatter points showing soft tissue shrinkage (mm) at Days 10, 21, and 30 for test and control sites.

**Figure 8:**
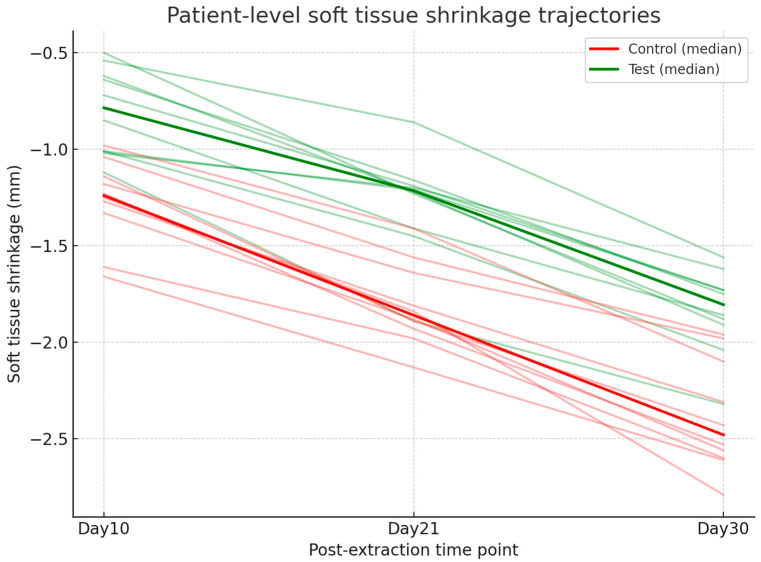
Line plot showing patient-level trajectories of soft tissue shrinkage (mm) at Days 10, 21, and 30 for test and control groups.

Digital analysis demonstrated consistently lower shrinkage in sockets treated with HPCTM compared with controls. At Day 10, the median paired difference (test–control) was 0.52 mm, with a Hodges–Lehmann estimate of 0.50 mm (95% CI: 0.29–0.62; Wilcoxon *p* = 0.0039). At Day 21, shrinkage remained reduced in the test group, with a median difference of 0.56 mm and an HL estimate of 0.54 mm (95% CI: 0.42–0.65; *p* = 0.002). At Day 30, the difference was sustained, with a median of 0.48 mm and an HL estimate of 0.54 mm (95% CI: 0.39–0.68; *p* = 0.002). Effect size estimates (rank-biserial correlation) indicated a moderate benefit at each time point. Figure 7 presents the distribution of shrinkage values, while Figure 8 illustrates patient-level trajectories, highlighting the consistent separation between test and control sites across the follow-up period.

### 3.2. Volumetric Bone Resorption

**Figure 9:**
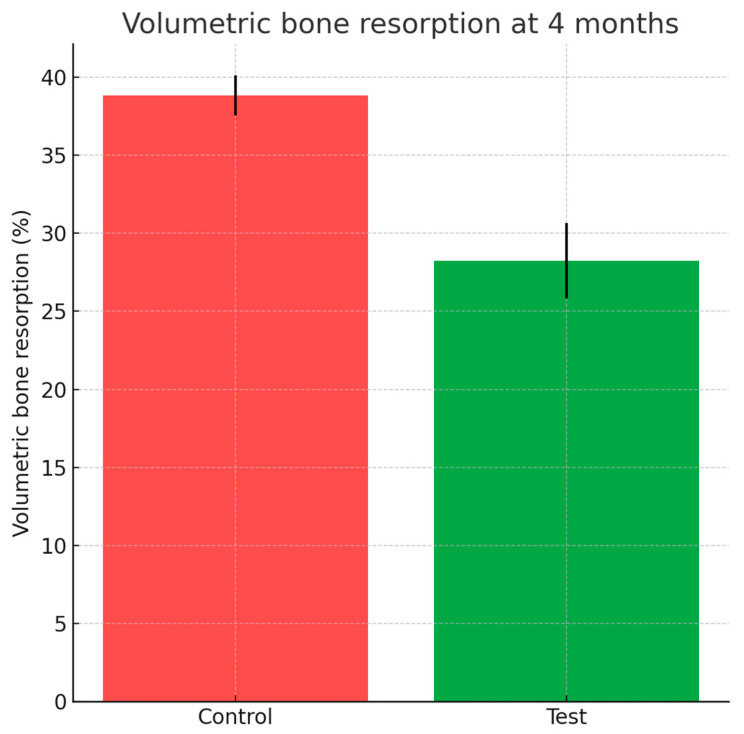
Bar chart with error bars depicting volumetric bone resorption (%) at 4 months for test and control groups.

Cone-beam computed tomography performed at four months revealed significantly greater dimensional stability in sockets treated with HPCTM. The mean volumetric reduction was 28.24 ± 2.43% at the test sites compared with 38.85 ± 1.29% for controls, corresponding to a preservation advantage of approximately 10.6% (Wilcoxon *p* = 0.019). These results are summarized in Figure 9.

### 3.3. Clinical Observations

Clinically, at the time of suture removal (10 days), inflammation of the socket periphery was seen at the control sites of all patients in addition to collapse and soft tissue shrinkage (Figure 1I); these features were not present at the test sites, where immature connective tissue covering the socket orifice over the grafted bone was seen with little gingival shrinkage (Figure 1J).

At 21 days post-op, the inflammation of the control sites was reduced, and the area of the socket orifice was filled with connective tissue (Figure 1K). This tissue occupied a larger area and was more developed and healthier at the test sites (Figure 1L). Moreover, the soft tissue circling the socket presented with voluminous cranial expansion at the test sites, whereas it had a caudal depression at the control sites.

At 30 days post-op, complete epithelialization was seen at the test and control sites, and the socket orifice zone was deemed larger at the test sites than at the control ones (Figure 1M,N).

## 4. Discussion

Simplifying implant-based rehabilitation by reducing post-extractive tissue loss remains a key objective in clinical practice [6]. ARP is widely employed to address this challenge; however, the loss of keratinized tissue and the need for secondary augmentation for implant placement remain unresolved [19]. In this context, the present study investigated the effect of supplementing allografts with flowable decellularized HPCTM on both soft and hard tissue stability following tooth extraction.

### 4.1. Interpretation of the Obtained Statistical Results

The findings demonstrated that the adjunctive use of HPCTM provided a measurable benefit in early healing. Digital analysis revealed a consistent reduction in soft tissue shrinkage at all follow-up intervals, with Hodges–Lehmann median differences of approximately 0.5 mm in favor of test sites, reaching statistical significance at Days 10, 21, and 30. In parallel, cone-beam tomography showed significantly lower volumetric bone resorption at test sites (28.2%) compared with controls (38.9%), corresponding to a preservation advantage. Together, these results suggest that HPCTM helps mitigate alveolar collapse by stabilizing both gingival contours and bone volume during the early phases of healing. These findings reject the null hypothesis of no within-subject difference between interventions for both the primary and secondary endpoints.

### 4.2. Biological Rationale

The biological explanation for these outcomes is consistent with established wound healing mechanisms. From a histological and cellular point of view, immediately after tooth extraction, the socket is filled with a blood clot that is gradually replaced in the following days with a provisional matrix rich in collagen and fibroblasts [20]. Biologically, it has been proven that the rebuilding of the lost tissue happens at this particular stage by means of fibroblasts and osteoblasts modulated by a series of growth factors [20].

In primary intention healing, those fibroblasts are protected from external stress by the extracellular matrix and can use the collagen fibers of the extracellular matrix as a scaffold to rapidly repair any trauma [21]. In contrast, following a tooth extraction where secondary intention healing is expected, the extracellular matrix is disturbed; as a response the fibroblasts activated by Transforming Growth Factor beta-1 (TGF-B1) start to secrete de novo collagen fibers to bridge the created defect and pull the wound edges together from the peripheries to the center, thus explaining the clinical soft tissue shrinkage [21].

For a long period of time, it was thought that the dimensional collapse after tooth extraction was mainly caused by bone resorption and the soft tissue following the bone levels. Current biological evidence suggests a different point of view; in fact, it was shown that the gingival tissue, mainly composed of collagen and elastin fibers, entraps endogenous growth factors, acts as a reservoir specifically for TGF-B1 and bone morphogenetic proteins [22,23], and can regulate matrix metalloproteinase-1 activity in wound healing [24]. As is known, the aforementioned growth factors and enzymes are directly implicated in bone homeostasis. Hence, one can clearly understand the extent of soft tissue’s role in alveolar ridge dimensional stability. In a post-extractive model, the sooner a mature connective tissue seal is created, the more collagen and elastin fibers there are; thus, less alveolar ridge collapse and bone resorption are seen. Currently, there is an abundance of collagen-based seals and matrices in the market; however, the main drawback is their uncontrollable resorption process and the non-biologically safe systematic addition of cross-linkers into their formulation [25,26]. Hence, there is a need for an actual seal that is integrated by the body as an actual extracellular matrix.

The HPCTM investigated in the present work is composed of a controlled and highly purified extracellular matrix composed of collagen types I and III in addition to laminin and fibronectin [17]. Those components, when mixed with the alveolar socket blood clot, provide a firm seal and scaffold essential for fibroblast migration and differentiation [17]. Additionally, the low immunogenicity of the created bone putty qualitatively improves and accelerates the healing process through the attraction of macrophages type II, an essential element of regeneration.

### 4.3. Comparison to Other Studies

Comparable studies on amnion–chorion membranes have shown similar bone preservation outcomes when such membranes are used in ridge preservation procedures [27,28], yet limited evidence exists regarding placental derivatives in soft tissue remodeling, especially in flowable formulations. The present findings therefore extend the current knowledge, suggesting that HPCTM, when combined with an allogeneic bone substitute, may improve both hard and soft tissue outcomes. This aligns with previous work on other extracellular matrix derivatives, such as hyaluronic acid, which also demonstrated a reduction in resorption and improved bone quality in a split-mouth design [18].

### 4.4. Limitations and Future Perspectives

This pilot study provides a proof of concept for the clinical use of flowable HPCTM in ridge preservation. While the results are promising, they should be interpreted within the limits of the small sample size. Larger-scale studies incorporating histomorphometric analysis and long-term implant outcomes are warranted to validate these preliminary observations and further define the role of placental-derived extracellular matrices in post-extraction management.

## 5. Conclusions

Adding flowable HPCTM to allografts helped preserve gingival contours and reduced bone loss after extraction. These preliminary results highlight the potential of HPCTM to improve ridge preservation, but larger clinical studies are needed to confirm its effectiveness.

## Figures and Tables

**Table 1:** Inclusion and exclusion criteria of the study.

Inclusion	Exclusion
Age 20–60 years Two hopeless teeth requiring extraction Teeth in the same morphological category (mono-, bi-, or multi-rooted) Teeth in different arch quadrants >50% of socket bone housing present Non-smoker	Active periodontal disease (Miller Class II or higher) Heavy metallic artifacts that distort CBCT (cone-beam CT) scans Poor oral hygiene and/or non-compliance Systemic diseases that interfere with bone metabolism Pregnant or lactating women Medication intake that interferes with bone metabolism Radiotherapy that involves the maxillofacial region

**Table 2:** Demographic data from this study.

Study Demographics	
Number of patients	10
Mean age	43, 19
Tooth type	
Maxillary Bicuspids/Maxillary molars	4/6
Mandibular Bicuspids/Mandibular molars	4/6

## Data Availability

The data presented in this study are available on request from the corresponding author due to ethical reasons.

## Abbreviations

The following abbreviations are used in this manuscript:

AAP	American Academy of Periodontology
ARP	Alveolar ridge preservation
CBCT	Cone-beam computed tomography
HPCTM	Human Placental Connective Tissue Matrix
HL	Hodges–Lehmann estimator
TGF-B1	Transforming Growth Factor beta-1
